# Giving Penetrable Remote-Control Ability to Thermoresponsive Fibrous Composite Actuator with Fast Response Induced by Alternative Magnetic Field

**DOI:** 10.3390/nano12010053

**Published:** 2021-12-25

**Authors:** Li Liu, Wenjing Song, Shaohua Jiang, Gaigai Duan, Xiaohong Qin

**Affiliations:** 1 Engineering Research Center of Technical Textiles, Ministry of Education, College of Textiles, Donghua University, Shanghai 201620, China; liliull@dhu.edu.cn (L.L.); swjing1217@163.com (W.S.); 2 Co-Innovation Center of Efficient Processing and Utilization of Forest Resources, College of Materials Science and Engineering, Nanjing Forestry University, Nanjing 210037, China; shaohua.jiang@njfu.edu.cn (S.J.); duangaigai@njfu.edu.cn (G.D.); 3 Fujian Key Laboratory of Eco-Industrial Green Technology, College of Ecology and Resources Engineering, Wuyi University, Wuyishan 354300, China

**Keywords:** composite actuator, alternative magnetic field, penetrable, electrospinning

## Abstract

An alternative magnetic field (AMF)-induced electrospun fibrous thermoresponsive composite actuator showing penetrable remote-control ability with fast response is shown here for the first time. The built-in heater of magnetothermal Fe_3_O_4_ nanoparticles in the actuator and the porous structure of the fibrous layer contribute to a fast actuation with a curvature of 0.4 mm^−1^ in 2 s. The higher loading amount of the Fe_3_O_4_ nanoparticles and higher magnetic field strength result in a faster actuation. Interestingly, the composite actuator showed a similar actuation even when it was covered by a piece of Polytetrafluoroethylene (PTFE) film, which shows a penetrable remote-control ability.

## 1. Introduction

Polymeric actuators that enable mechanical motions driven by stimulus from surroundings would have a great potential for soft robotic [1], sensor [2,3] and biomedical applications [4,5]. They mostly rely on anisotropy created across the thickness in the shape of layer structure to generate mechanical behaviors [6,7]. Actuation from an anisotropic structure is a very common phenomenon in daily life [8,9,10,11]. A piece of paper bends or curls on a calm water surface due to the differential swelling behavior in the thickness direction caused by water diffusion [12,13]. To make actuation happen, smart materials are involved in most cases, which can be altered by stimulus, such as temperature [14,15], pH [16,17], light [18], humidity [19], etc. [20,21].

Among them, the thermoresponsive ones made by Poly(N-isopropylacrylamide) (PNIPAM) have attracted a lot of scientists’ attention, because they possess a nature such that the trigged temperature is at 32 °C, close to body temperature, which made it one of the candidates for biomedical applications [22,23]. Noteworthily, the actuation process of thermoresponsive actuator is inevitably accompanied by a heat transfer, which plays a significant role in the sensitivity [24]. However, currently, most thermoresponsive actuations developed so far mainly reply on the outer heat source [25]. In such an outer heat source system, it results in a lot of energy loss when the heat moves from the heat source to the target of actuator through the medium, such as water [26]. It causes a lot of heat loss, since only the medium/water around the active layer of PNIPAM directly contributes to the actuation and the remaining amount of water occupies the extra heat that should belong to the actuator. This is a kind of passive response way limiting its applications. To overcome this point, a new concept of built-in heater has been proposed recently [27,28]. Wu et al. fabricated a near infrared (NIR)-driven actuator by incorporating photothermal reduced graphene oxide (rGO) into PNIPAM. This actuator can perform precise actuation control depending on the position of the NIR spot [29]. The basic concept is to create a built-in heater in the actuator by combining photothermal components, thus offering a possibility of realizing localized heating instead of gaining heat from an outer heat source [30]. Nonetheless, although it is non-contact actuation and can be applied in some particular fields by employing this kind of NIR-induced localized heating model, it will lose efficacy when the actuator is covered by an opaque medium obstructing the passage of NIR. Instead, Wang et al. embedded magnetocaloric Fe_3_O_4_ nanoparticles, rather than rGO, into PNIPAM film, forming a film actuator that achieves a curvature of 0.2 mm^−1^ in more than 200 s [31]. Similarly, Xie et al. added Fe_3_O_4_ nanoparticles into epoxy, fabricating a shape memory polymer (SMP) and used AMF to control the actuation [32]. Despite the fact that these actuators showed remote-control, they did not pay attention to the penetrable remote-control ability. Additionally, those actuations were slow because of the compact structure of bulk film suppressing the heat transfer among the components. In our previous study [25,33], it was found that electrospinning is a facial technique to produce fibers [34]; it has also been demonstrated that the electrospun fibrous thermoresponsive actuator can facilitate the actuation and improve the sensitivity owing to the porous structure [25,33]. Therefore, this prompted us to explore a new thermoresponsive actuator possessing penetrable remote-control ability with fast actuation [35].

Herein, a composite thermoresponsive actuator with multifunctional actuation upon an alternative magnetic field (AMF) was fabricated. Fe_3_O_4_ nanoparticle-embedded thermoplastic polyurethane (TPU) film and a photo-crosslinkable electrospun fibrous P(NIPAM) mat formed the bilayer actuator. The porous structure of the fibrous P(NIPAM) mat guarantees a high-efficiency heat transfer generated from the TPU side, thus causing a fast response. It turns out that the bending curvature can reach 0.4 mm^−1^ in 2 s and that a higher loading amount of Fe_3_O_4_ and higher magnetic strength generate higher temperature, giving rise to faster actuation. This actuator can still show a stable and fast remote-control actuation, even when covered by a PTFE film upon (AMF), due to the penetrability of AMF. This electrospun fibrous mat-based AMF-induced approach may pave a new way for conducting a penetrable control remote-control and fast actuation.

## 2. Experimental Section

### 2.1. Materials

Azobisisobytyronitrile (AIBN) (Acmec, Shanghai, China), 4-hydroxybenzophenone (ABP) (Acmec, Shanghai, China), N-isopropylacrylamide (NIPAM) (TCI, Shanghai, China), dioxane (Sinopharm, Shanghai, China), thermoplastic polyurethane (TPU) (Desmopan 9095AU DPS300, Covestro, Shanghai, China), surfactant (TNDDIS, Timesnano, Shanghai, China), N,N′-dimethylformamide (DMF) (99.8%, Sinopharm, Shanghai, China), and Fe_3_O_4_ nanoparticles (99.5%, 20 nm, Acmec, Shanghai, China) were used as received. The synthesis of photo cross-linkable Poly(N-isopropylacrylamide-co-4-acryloyloxybenzophenone) (P(NIPAM-ABP)) was performed as previous described [25].

### 2.2. Fabrication of AMF-Induced Fibrous Composite Actuator

**Figure 1:**
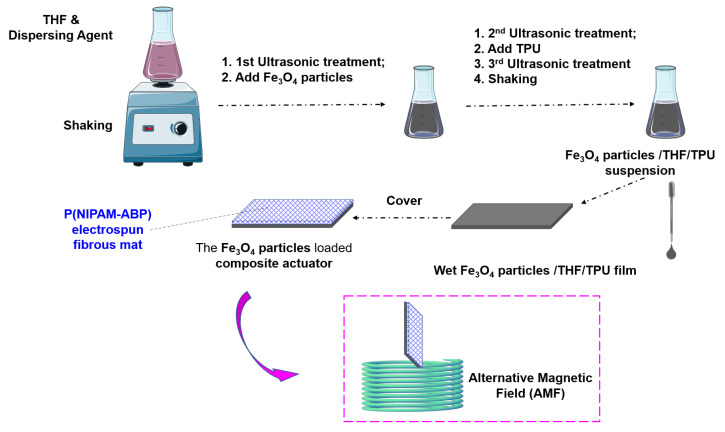
Schematic of the composite actuator fabrication process.

The fabrication of the induced fibrous composite actuator can be broken down into 3 steps. As illustrated in Figure 1, briefly, a wet TPU/Fe_3_O_4_ composites film was covered by a P(NIPAM-ABP) electrospun fibrous mat. Firstly, the P(NIPAM-ABP) electrospun fibrous mat was fabricated from a 40 wt% P(NIPAM-ABP)/DMF solution under 17 kV with a feeding rate at 1.2 mL/h, followed by the photo cross-linked by a UV light (Benmi, 365 nm) for 1 h for both sides. Secondly, the Fe_3_O_4_ nanoparticles suspension was prepared by adding 0.03 g Fe_3_O_4_ nanoparticles into a mixed solution of 10 mL THF and 0.12 g surfactant, followed by ultrasonic treatment for 120 min. After that, 0.6 g TPU was transferred into the mixture and shook for 24 h until a homogeneous suspension was iformed. Finally, 0.5 mL suspension prepared above was homogeneously dropped on a piece of glass slide forming the TPU/Fe_3_O_4_ composites film, and the cross-linked P(NIPAM-ABP) electrospun fibrous mat was used to cover it immediately. Consequently, the AMF-induced fibrous composite actuator was created after it is dry.

### 2.3. Characterizations

A scanning electron microscope (SEM) (DXS-10ACKT, Tianjin, China) and a field emission scanning electron microscope (FESEM) (SU8010, Hitachi, Shanghai, China) were used to check the morphology of TPU film and P(NIPAM-ABP) fibrous mat, and to identify the distribution of Fe_3_O_4_ particles in TPU film. A 3.0 nm layer of platinum (Pt) was sputtered before scanning, to improve the conductivity of the sample. The temperature change upon AMF was determined by an infrared (IR) camera (PTi 120, Fluke, Shanghai, China) and the actuation process was captured by a cellphone (Nova7se, Huawei, Shanghia, China). The dimensions of the actuator samples were all set at 20 mm × 10 mm when conducting the actuation. The AMF device (SPG-06A-III) was purchased from Shuangping Power Supply Technology Company Ltd (Shanghai, City). The porosity of the electrospun fibrous mat was calculated by the following equations:

(1) $$\rho_{mat}=\frac{m_{mat}}{a_{mat}\times t_{mat}}$$

(2) $$\rho_{film}=\frac{m_{film}}{a_{film}\times t_{film}}$$

(3) $$P=\left(1-\frac{\rho_{mat}}{\rho_{film}}\right)\times 100\%$$

where *ρ_mat_* and *ρ_film_* are the density of the fibrous mat and the corresponding film, respectively; *a_mat_* and *a_film_* are the area of fibrous mat and the corresponding film, respectively; *t_mat_* and *t_film_* are the thickness of fibrous mat and the corresponding film, respectively.

## 3. Results and Discussion

### 3.1. Morphology of Electrospun Fibrous Mats

**Figure 2:**
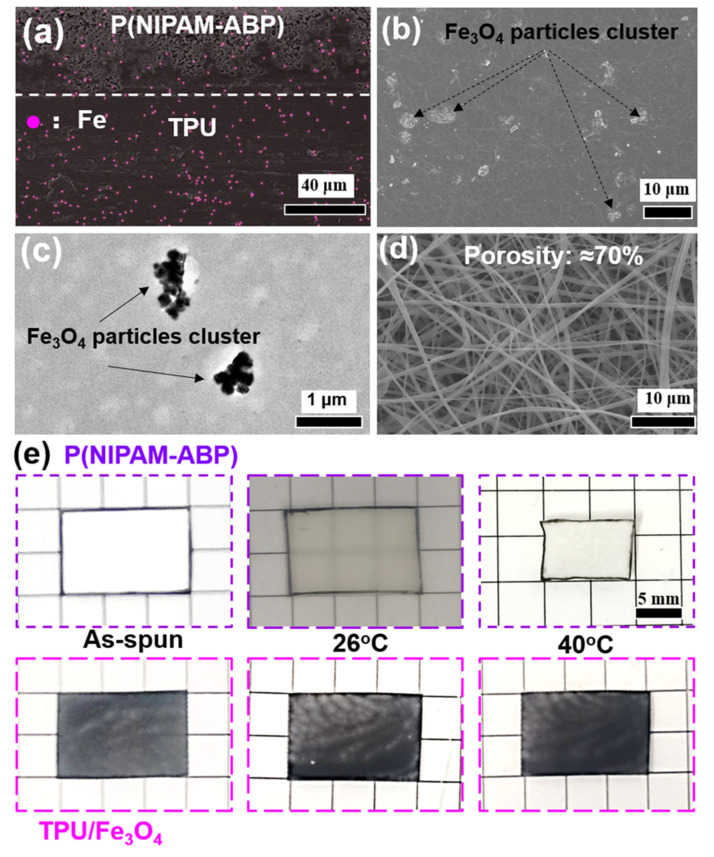
Morphology of the composite actuator. (**a**) The EDS mapping of the cross-section of the actuator, the pink dots indicate the Fe element; the SEM and TEM images of the TPU/Fe_3_O_4_ particles side were shown in (**b**,**c**), respectively; (**d**) the SEM image of the P(NIPAM-ABP) fibrous mat; (**e**) size changes of P(NIPAM-ABP) electrospun fibrous mat (white) and TPU/Fe_3_O_4_ film (black) in water with different temperatures.

To experimentally validate the concept that a built-in heater in the actuator can provide penetrable remote-control with fast actuation upon AMF, magnetothermal Fe_3_O_4_ nanoparticles were embedded into the actuator as described. To verify the existence and distribution of Fe_3_O_4_ particles in the actuator, TEM, SEM and corresponding EDS mapping were conducted. As presented in Figure 2, the SEM and TEM micrographs evidence that the Fe_3_O_4_ particles have been embedded into the TPU film. The EDS mapping of the cross-section in Figure 2a confirms the Fe composition coming from Fe_3_O_4_ particles and displays a uniform distribution of Fe_3_O_4_ particles across the thickness. As a result of the porous structure, some suspension of TPU/Fe_3_O_4_ particles was penetrated into the fibrous mat side creating a strong adhesion between the film and the fibrous mat without any delamination. The Figure 2b, c shows that Fe_3_O_4_ particles are in the form of clusters distributed in the TPU film due to the high loading amount of the particles. These particles embedded into the film will be turned into numerous built-in heaters offering the energy for the contraction of P(NIPAM-ABP) upon AMF, which will be discussed later in the next section. The uniform distribution of Fe_3_O_4_ particles in the TPU film and the high porosity of the fibrous mats at 70% (Figure 2d) can guarantee a homogeneous and fast heat transfer, which contribute to the stable and fast actuation.

As illustrated in Figure 2e, the TPU/Fe_3_O_4_ composite did not show any size change in water with temperatures. On the other hand, the P(NIPAM-ABP) fibrous mat did not show an obvious change in dimension at 26 °C, since no obvious phase separation occurs around its lower critical solution temperature (LCST) of 27 °C. However, as the temperature increased to 40 °C, the P(NIPAM-ABP) fibrous mat showed an isotropic shrinkage by about 38% in both length and width, in comparison to the dry state and the one at 26 °C. This is because of the coiled conformation of P(NIPAM-ABP) polymer chains and the relaxation of the electrospun fibers [33].

### 3.2. Effect of Fe_3_O_4_ on Temperature upon Alternative Magnetic Field

**Figure 3:**
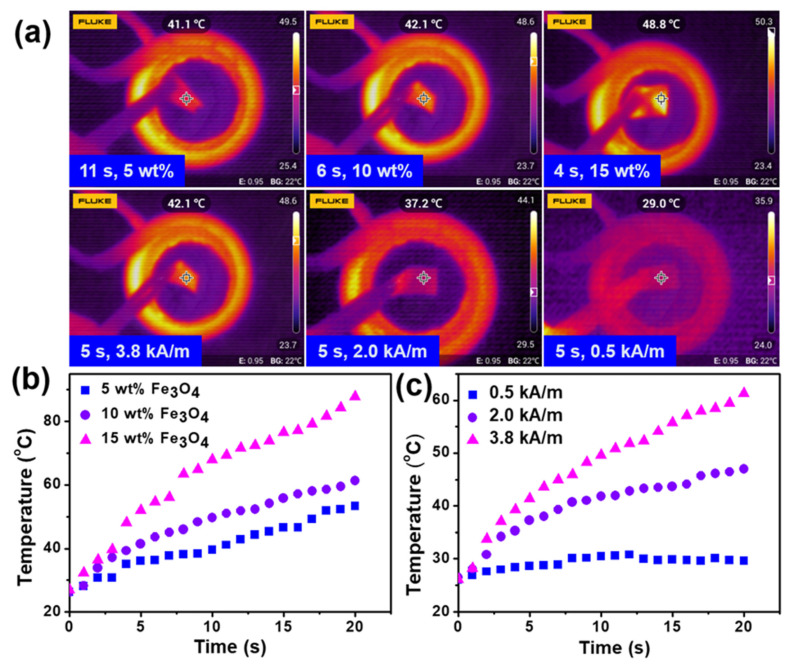
Temperature change profile for the dry composite actuators. (**a**) IR camera images; (**b**) the temperature changeof TPU surfaceas a function of Fe_3_O_4_ particles loading amount; (**c**) the exposure time-dependent TPU surface temperature of the actuator with 10 wt%-loaded Fe_3_O_4_ particles upon different magnetic field strength.

The heating property of the dry composite actuator was investigated by subjecting them to 600 kHz AMF. As shown in Figure 3, the TPU surface temperatures of the composite actuator were plotted with the exposure time. An IR camera was used to monitor the surface temperature change (Figure 3a). These samples have three Fe_3_O_4_ particles concentrations with respect to the amount of TPU, respectively. Upon AMF, the Fe_3_O_4_ particles in the TPU film of the actuator absorbed the energy from the AMF and converted them into heat, thereby resulting in a sharp increase in TPU surface temperature. As illustrated in Figure 3b,c, the temperature of the TPU surface was increased from 26 °C to 30 °C within 2 s. The temperature is proportionate to the loading amount of the Fe_3_O_4_ particles. Fe_3_O_4_ nanoparticle can induce fast energy conversation into heat upon AMF. Consequently, the more loading amount of Fe_3_O_4_ nanoparticles in the sample, the more conductive to the heat generating, thus causing faster temperature increase. Moreover, the samples with a higher loading amount can always achieve a higher temperature at a specific exposure time (Figure 3b). In addition, the effect of the magnetic field strength was studied as well. The Figure 3c manifested that the magnetic field strength also plays a significant role in the temperature increasement. Higher magnetic field strength results in higher temperature, but under a low magnetic field strength of 0.5 kA/m, the temperature of the actuator rises slowly and finally becomes constant at around 30 °C. Please note that the tweezer used in the experiments was made from plastic, which is insensitive to AMF.

### 3.3. Mechanism and Actuation Process

The paper curl phenomenon described previously in the introduction prompted us to create an actuator, possessing anisotropy across the thickness, by constructing a bilayer structure. This bilayer structure comprises a passive layer of TPU/Fe_3_O_4_ composite, which is not able to show size change in water with different temperature and an active layer of P(NIPAM-ABP), which allows for size change upon temperature change due to its thermoresponsive property, as shown in Figure 2e.

**Figure 4:**
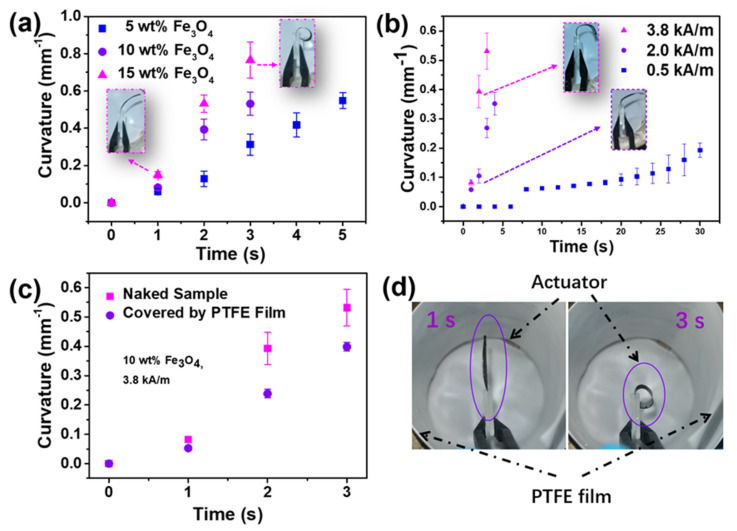
Curvature change profile for different Fe_3_O_4_ loading amount actuators upon different magnetic field strength and the penetrable remote−control ability of the actuator. (**a**) the exposure time-dependent curvature of the composite actuator with different Fe_3_O_4_ loading amount; (**b**) the exposure time−dependent curvature of the composite actuator with 10 wt%−loaded Fe_3_O_4_ particles upon different magnetic field strength; (**c**) the difference of curvature change profile of the naked sample and the one covered by a PTFE film indicating the penetrable remote−control ability, and (**d**) the image of PTFE-film covered sample.

Therefore, a compromise, in the form of bending, as a result of the competition between the passive layer of TPU/Fe_3_O_4_ film and the active layer of the P(NIPAM-ABP) fibrous mat is created. When a wetted composite actuator is triggered by AMF, the generated heat resulting from Fe_3_O_4_ particles in the actuator causes the contraction of P(NIPAM-ABP) fibers, thus performing bending behavior. It is worth noting that, the heat transfer by this approach is different from the one by traditional actuator. In this Fe_3_O_4_ particles-embedded actuator, the heat resource is located inside the actuator and the generated heat from this built-in heater is transferred to the P(NIPAM-ABP) fibrous layer, mainly through the water/vapour among fibers. It implies that the heat is only transferred inside the actuator and can reduce the actuation’s dependence on the environment. Contrarily, the actuation of the traditional thermoresponsive actuators is highly dependent on the outer heat source, and the medium between the outer heat source and the actuator consumes most of the heat, lowering the efficiency. It is highly dependent on the temperature change outside the actuator [36,37]. Furthermore, the efficiency of heat transfer can benefit from the high porosity of the fibrous mat rather than the compact structure of film. Therefore, as illustrated in Figure 4, the actuator fabricated by our new approach exhibits a fast actuation that allows the bending curvature to reach 0.4^−1^ mm in 2 s upon AMF, but the similar pure film one takes more than 250 s to achieve the same curvature [31]. Both higher loading amount of particles and higher magnetic field strength contribute to a faster actuation (Figure 4a,b), and, noteworthily, a sharp rise in curvature occurs once the temperature reaches and exceeds 27 °C, irrelevant of loading amount and magnetic field strength (Figure 3b,c and Figure 4). This is consistent with the transition of thermoresponsive polymer P(NIPAM-ABP) around its lower critical solution temperature at 27 °C [33].

### 3.4. Penetrable Remote-Control Ability of the Composite Actuator

Generally, it is possible for NIR-triggered actuators to proceed non-contact actuation, but they will loss efficacy once the passage of NIR is obstructed. To some extent, it will limit its application in some specific field. Correspondingly, one of the unique characteristics of this composite actuator is that the actuation can still be non-contact manipulated even when it is covered by an opaque medium. As shown in Figure 4c,d, the composite, which is covered by a piece of PTFE film in the AMF, can still show a similar actuation with the naked sample, but a little bit lower curvature due to the penetrability of AMF. Therefore, there is a possibility that the introduction of AMF can give the actuator a penetrable remote-control ability, which can expand their potential.

## 4. Conclusions

An alternative magnetic field (AMF)-triggered thermoresponsive electrospun composite actuator with penetrable remote-control ability and fast response was demonstrated. It can achieve a curvature at 0.4 mm^−1^ in 1 s and still shows a similar actuation even when the actuator was covered by a piece of PTFE film. The actuation led to bending movement with temperature affected by the loading amount of Fe_3_O_4_ particles and the magnetic field strength. The built-in heater in the composite actuator, and the inherent porosity of the electrospun P(NIPAM-ABP) fibrous mat can facilitate the heat transfer among the fibers, thus causing fast actuation. The nature of AMF, that of penetrability, can provide the actuator with penetrable remote-control ability. This approach is simple and provides a possibility to offer an actuator a penetrable remote-control ability with fast response and probably expand their potential in biomedical engineering and soft robotics.

## Data Availability

The data presented in this study are available on request from the corresponding author.
